# Re-engineering segment 8 facilitates generation of a versatile live-attenuated influenza A virus vector platform for secretory protein delivery

**DOI:** 10.1128/jvi.00347-26

**Published:** 2026-04-21

**Authors:** Soner Yildiz, Sara S. El Zahed, Fernando Villalón-Letelier, Qian Wang, Sowmya Yelleswarapu, Gloria Dawodu, Kaijun Jiang, Raveen Rathnasinghe, Umut Karakus, Rocio Seoane, Sara Cuadrado-Castano, Adolfo García-Sastre

**Affiliations:** 1 Department of Microbiology, Icahn School of Medicine at Mount Sinai 200769, New York, New York, USA; 2 Global Health and Emerging Pathogens Institute, Icahn School of Medicine at Mount Sinai 5925, New York, New York, USA; 3 Graduate School of Biomedical Sciences at the Icahn School of Medicine at Mount Sinai 5925, New York, New York, USA; 4 Department of Pathology, Molecular and Cell-Based Medicine, Icahn School of Medicine at Mount Sinai 5925, New York, New York, USA; 5 Icahn Genomics Institute, Icahn School of Medicine at Mount Sinai 5925, New York, New York, USA; 6 Precision Immunology Institute (PRIISM), Icahn School of Medicine at Mount Sinai, New York, New York, USA; 7 Division of Infectious Diseases, Department of Medicine, Icahn School of Medicine at Mount Sinai 377569, New York, New York, USA; 8 Tisch Cancer Institute, Icahn School of Medicine at Mount Sinai, New York, New York, USA; University of North Carolina at Chapel Hill, Chapel Hill, North Carolina, USA

**Keywords:** IL-15, IL-2, secretory protein delivery, ΔNS1 vectors, influenza A virus

## Abstract

Influenza A viruses (IAVs) lacking the nonstructural protein 1 (NS1) gene (IAV-ΔNS1) are attenuated both *in vitro* and *in vivo,* yet retain replicative capacity in interferon signaling-deficient systems. To date, several efforts have been pursued to expand its functionality by incorporating transgenes in lieu of the NS1 gene. However, such vectors require complex segment designs, retain residual NS1 peptide stretches, or display suboptimal cytokine secretion, largely due to the constraints related to the partial nuclear export protein (NEP) open reading frame (ORF) upstream of the transgene integration site. Here, we describe a novel segment 8 design that can accommodate a transgene, where the complete NEP ORF is positioned downstream of the splicing acceptor site, thereby mitigating previous genetic constraints. Using this strategy, we successfully generated a recombinant IAV-ΔNS1 virus carrying the human interleukin 2 (IL-2) gene, inducing robust secretion of bioactive IL-2 upon infection. The vector demonstrated high replicative capacity, achieving titers comparable to wild-type viruses in MDCK cells expressing NS1 protein, and exhibited genetic stability over 10 successive passages. Importantly, infection induced notable viral antigen expression and high levels of bioactive IL-2 secretion in interferon-competent systems, such as MDCK, A549, and HEK293T cells *in vitro* and in mice upon pulmonary delivery *in vivo*. Furthermore, the transgene was easily swapped with a diverse array of genes encoding human IL-15, nanoluciferase, or fluorescent proteins such as miniSOG and miRFP670nano, highlighting the versatility of the platform.

**IMPORTANCE:**

IAV-ΔNS1 vectors are promising vaccine platforms that elicit strong immune responses with a good safety profile. However, integration of immune-stimulatory cytokines into vector design to boost immunogenicity has been technically challenging. In this study, we developed a genetically re-engineered segment 8 design that overcomes prior limitations due to design complexity or vector efficiency, enabling high-level expression of the transgene, i.e., human interleukin 2 and other diverse proteins. The platform retains strong replicative capacity in permissive systems and remains genetically stable, making it suitable for scalable vaccine or therapeutic development. By improving both the flexibility and functionality of IAV-ΔNS1 vectors, our work advances the utility of influenza A viruses as customizable tools for vaccine delivery, immune modulation, and therapeutic applications.

## INTRODUCTION

Influenza A virus (IAV) is the etiological agent responsible for influenza, a respiratory illness in humans with mild to severe symptoms, commonly referred to as the flu. Belonging to the Orthomyxoviridae family, its viral genome consists of eight single-stranded RNA segments of negative polarity (1, 2). Each segment is transcribed and replicated by the viral polymerase complex to produce viral proteins and genomic RNA required for propagation (2).

The mRNA transcribed from segment 8 encodes at least two non-structural proteins: non-structural protein 1 (NS1) and nuclear export protein (NEP) (3–5). NS1 is a key viral protein that promotes immune evasion primarily by suppressing host innate immune signaling and interfering with host RNA processing and mRNA export (6–9). On the other hand, NEP is required for optimal viral RNA replication and facilitates the nuclear export of viral ribonucleoproteins, thereby enabling efficient viral assembly and production of infectious progeny (10, 11). The unspliced segment 8 transcript encodes the NS1 protein, while NEP is translated from the alternatively spliced variant processed by the host splicing machinery (3–5). Due to suboptimal splicing regulatory signals, splicing of segment 8 mRNA yields an approximately 10:1 ratio of NS1 to NEP protein (4, 5, 12), enabling temporal regulation of NEP levels that coordinates the switch from viral RNA synthesis to assembly (10, 11).

Plasmid-based reverse genetics systems have facilitated the generation of recombinant influenza viruses (13–15) and the rational design of live-attenuated vaccine prototypes (16, 17), such as IAVs with partial or complete deletions of the NS1 protein (IAV-ΔNS1) (18–20). Lacking a key virulence factor, IAV-ΔNS1 vectors are attenuated both *in vitro* and *in vivo*, yet retain replicative capacity in interferon (IFN) signaling-deficient systems (21). Intranasal administration of IAV-∆NS1 vectors has been shown to induce protective immunity in mice, pigs, horses, and macaques (18–20) and has demonstrated encouraging results in clinical testing within the last decade as a live-attenuated vaccine (22–26). These vectors are considered strong candidates for next-generation influenza vaccines, as they provide protective immunity at the site of natural infection, are safe for clinical use, and offer flexibility in production. Consequently, IAV-ΔNS1 vectors have been widely explored as a promising platform for live-attenuated influenza vaccines.

To date, several strategies have been pursued to incorporate reporter genes or immunomodulatory cytokines in lieu of the NS1 gene to enhance the functionality of the ΔNS1 vector platforms (27–31). These include designs in which transgenes are expressed as self-cleaving peptide fusions to NEP or placed within the intronic region between the splicing regulatory signals and expressed using stop-start translation signals while maintaining the segment 8 structure. However, current strategies do not support optimal transgene integration and expression without compromising viral fitness or disrupting the temporal dynamics of NEP protein. Additionally, post-translational control and intracellular trafficking of the transgene are often hindered due to protein tethering or weak translation initiation, particularly for secretory transgenes or those requiring N-terminal processing. These limitations stem largely from the requirement to preserve the partial NEP ORF upstream of the transgene integration site, which constrains the genetic frame and limits design flexibility. Therefore, there is a clear need for a more versatile and streamlined strategy capable of efficiently supporting the expression of the transgene with minimal impact on viral fitness.

In this study, we describe a novel, re-engineered segment 8 design in which the complete NEP ORF is re-positioned downstream of the native splicing acceptor site, thereby mitigating previous genetic constraints and enabling flexible transgene integration. In summary, here, we developed a novel, genetically stable, and robust IAV-ΔNS1 vector capable of efficiently expressing a secretory protein or other reporter genes.

## RESULTS

### Modified segment 8 enables robust IL-2 secretion while preserving viral fitness and stability

**Fig 1:**
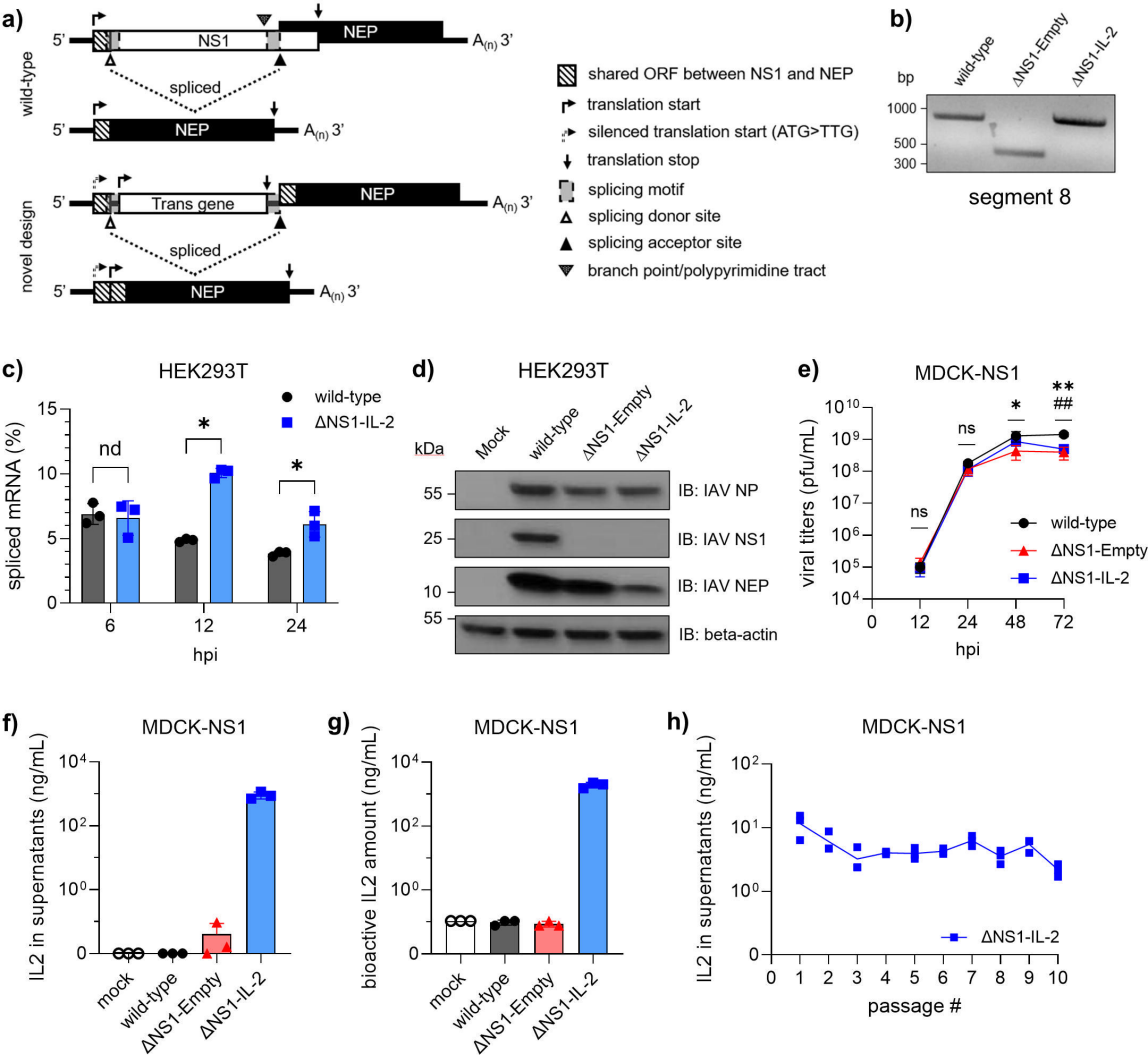
Re-engineered segment 8 enables seamless integration of the human IL-2 ORF into the IAV-ΔNS1 vector backbone. (**a**) Schematic representation of alternative splicing for influenza A virus mRNA encoded by wild-type or re-engineered segment 8. Icon descriptions are provided in the figure. Not to scale. (**b**) Representative agarose gel image showing segment 8-specific RT-PCR products amplified from viral RNAs for the indicated viruses. (**c**) Ratios of spliced versus total segment 8 mRNA copy numbers from HEK293T cells infected with viruses carrying wild-type or ΔNS1-IL-2 segment 8 at an MOI of 1, quantified by qRT-PCR, at the indicated time points. Representative data from two independent experiments are depicted as mean ± SD (*n* = 3). (**d**) Western blot images acquired from lysates of HEK293T cells infected with the indicated viruses or mock controls at 24 hours post-infection. A representative image is shown. (**e**) Multi-cycle growth curve analysis for the indicated viruses in MDCK-NS1 cells. Data are depicted as mean ± SD (*n* = 3). Representative data from two independent experiments are shown. * and # indicate statistically significant differences between wild-type and vector control, or between wild-type and the IL-2-carrying vector, respectively. IL-2 ELISA (**f**) and IL-2 bioactivity assay (**g**) results for supernatants collected from MDCK-NS1 cells infected with indicated viruses at an MOI of 1 at 24 hpi. Representative data from two independent experiments are depicted as mean ± SD (*n* = 3). (**h**) IL-2 ELISA results for supernatants collected from consecutive blind passages of ∆NS1-IL-2 viruses on MDCK-NS1 cells. Data are depicted as mean ± SD (*n* = 3). Student’s *t*-test was used to test for statistical significance unless otherwise stated. *P* < 0.05. bp, base pair; hpi, hours post-infection; IB, immunoblot; kDa, kilodalton; pfu, plaque-forming unit; mL, milliliter; and ng, nanogram.

To overcome genetic limitations inherent to the segment structure, we re-engineered segment 8 by decoupling the NEP ORF from the upstream NS1 ORF (Fig. 1a). Briefly, the transgene was inserted into the intronic region in lieu of NS1 ORF while preserving the splicing regulatory elements. The full-length NEP ORF, including the N-terminal 10 amino acids, was re-positioned downstream of the native splice acceptor site, creating genetic flexibility in defining the translation initiation for the transgene. Accordingly, the unspliced mRNA is designated to encode only the transgene ORF independently, whereas the spliced variant expresses NEP without relying on the upstream genetic elements (Fig. 1a).

The construct was based on the A/Puerto Rico/8/1934 (PR/8) segment 8 backbone. Nucleotides 1–64 on the positive strand, which include the 3′ packaging signal and native splice donor motif, were synthesized via primer annealing. The native NS1 start codon (ATG) was mutated to TTG to ensure translation initiation at the downstream transgene ORF. Separately, the downstream region containing the splice acceptor motif, the complete NEP ORF, and 5′ packaging signals, in the given order, was assembled through a series of PCRs using PR/8 segment 8 as a template. The human IL-2 gene, amplified from the commercially available plasmid (RC210013, Origene) and flanked with homologous sequences, was fused to the adjacent segments by nested PCR and cloned into a pDZ vector.

Next, we used reverse genetics to rescue PR/8 viruses in a 7+1 system using plasmids encoding either the wild-type or the re-engineered segment 8 containing human IL-2 (ΔNS1-IL-2), or an otherwise identical construct lacking the transgene (ΔNS1-Empty). Incorporation of the modified segments into the viral genome was confirmed by segment-specific RT-PCR, followed by size evaluation via agarose gel electrophoresis (Fig. 1b), and the sequences were verified by Sanger sequencing.

To evaluate the splicing efficiency of the modified segment, HEK293T cells were infected at a multiplicity of infection (MOI) of 1 with either wild-type or ΔNS1-IL-2 viruses. Total RNA was extracted at 6, 12, and 24 hours post-infection, and splice variants were quantified relative to total segment 8 mRNA by RT-qPCR. The virus carrying the re-engineered segment showed a significant increase in spliced mRNA levels compared to the wild-type virus at 12 and 24 hours post-infection, whereas similar levels were observed at the early time point, 6 hpi (Fig. 1c). Next, Western blot analysis of HEK293T cells at 24 hours post-infection demonstrated that the NEP/NP signal ratio in ΔNS1-Empty infected cells was comparable to that in wild-type virus-infected cells (wild-type: 1.6; ΔNS1-Empty: 1.9; Fig. 1d). Interestingly, the ratio in ΔNS1-IL-2-infected cells was reduced by approximately half compared with the vector control (ΔNS1-IL-2: 0.8), despite similar NP expression levels (Fig. 1d). These findings suggest that the re-engineered construct largely mimics wild-type splicing and NEP expression, with notable deviations over the course of infection, suggesting that the presence of NS1 protein, intron length, or sequence composition may affect splicing efficiency. As expected, neither ΔNS1-Empty nor ΔNS1-IL-2 viruses expressed detectable NS1 protein (Fig. 1d).

To assess the replication kinetics in a multi-cycle infection setting, MDCK-NS1 cells were infected with wild-type, ΔNS1-IL-2, or ΔNS1-Empty viruses at an MOI of 0.02. Viral infectious particles in supernatants collected at various time points, i.e., 12, 24, 48, and 72 hours post-infection, were quantified by standard plaque assays. Data demonstrated that re-engineered viruses replicated to titers comparable to those of the wild-type control (10^8^–10^9^ pfu/mL) with only minor differences observed at 48 and 72 hours post-infection (Fig. 1e), indicating that the re-engineered segment had minimal impact on viral replication.

We next assessed the capacity of the vector for cytokine delivery and secretion. To this end, MDCK-NS1 cells were infected with the ΔNS1-IL-2, the vector control (ΔNS1-Empty), or the wild-type virus at an MOI of 1. Supernatants were harvested at 24 hours post-infection, heat-inactivated, and analyzed by enzyme-linked immunosorbent assay (ELISA) for secreted IL-2 levels and by reporter assay (HEK-Blue IL-2/IL-15 reporter cells) for IL-2 bioactivity. The ΔNS1-IL-2 virus induced robust secretion of bioactive IL-2, with concentrations reaching the microgram per milliliter range (Fig. 1f and g), while control viruses (wild-type and ΔNS1-Empty) showed no detectable IL-2 expression. Of note, when IL-2 was expressed as a 2A-linked fusion upstream of the NEP ORF (Fig. S1a), a previously used strategy for reporter transgene expression in ΔNS1 viruses (31), no IL-2 secretion was detected in infection supernatants (Fig. S1b), suggesting that direct fusion may interfere with the post-translational processing and/or secretion of IL-2.

Maintaining the structure and sequence identity of a foreign gene within the influenza A virus vector is inherently challenging due to the high mutation rates of viral RNA polymerases and the segmented nature of the genome. Hence, to assess the genetic stability of our vector design, the ΔNS1-IL-2 virus was serially passaged in MDCK-NS1 cells for 10 blind passages, with each infection lasting for 72 hours. Supernatants were collected at each passage, and IL-2 levels were measured as a surrogate marker for the transgene integrity. As shown in Fig. 1h, IL-2 secretion remained robust across all passages, with minor fluctuations. Surprisingly, IL-2 levels in the supernatants at 72 hpi (Fig. 1h) were approximately 2-log lower than those measured at 24 hpi in single-cycle infections (Fig. 1f). Consistent with our time-course data, vector-induced IL-2 secretion peaked at 24 hpi and declined thereafter (data not shown), which explains the lower concentrations observed at 72 hpi. To verify that this functional readout accurately reflected transgene integrity, viral RNA from the 10th passage was sequenced using Oxford Nanopore Technologies. While two single nucleotide polymorphisms (SNPs) were identified in the HA and NP segments, no mutations were detected within the engineered segment across three independent replicates (Table S1), underscoring the genomic stability of the construct.

In summary, our data show that the novel vector design is capable of high-level transgene expression and secretion, while preserving splicing fidelity, replication competence, and genomic stability.

### Canonical branch point is dispensable in the re-engineered segment 8

Pre-mRNA splicing relies on a polypyrimidine tract and a branch point to facilitate U2 small nuclear ribonucleoprotein recruitment and lariat formation (32). In IAV, the canonical branch point within segment 8 has been mapped to an adenine located approximately 20 nucleotides upstream of the splicing acceptor site (5, 33). In our re-engineered segment 8 construct, the canonical branch point was absent, and the polypyrimidine tract was only partially retained. We hypothesized that reintroduction of these elements might further enhance splicing efficiency, thereby improving transgene expression as well as replicative capacity.

To test this, we rescued recombinant viruses carrying two different segment 8 designs: one encoding IL-2 as described in Fig. 1a (ΔNS1-IL-2), and another construct containing an additional 12 nucleotides upstream of the splice acceptor site to restore both the native branch point and the complete polypyrimidine tract (ΔNS1-IL-2 + BP). Expectedly, restoration of these canonical elements in the viral vector resulted in a modest but significant increase in splicing efficiency at 12 and 24 hpi in HEK293T cells (Fig. S2a), as determined by RT-qPCR. Surprisingly, infection in HEK293T cells with either virus led to similar levels of NP and NEP expression (NEP/NP ratio: 0.6 and 0.5; Fig. S2b). However, viral replication was slightly reduced at 12 hpi for the virus containing the branch point in MDCK-NS1 cells and later became comparable at subsequent time points (Fig. S2c). Similarly, both viruses exhibited similar levels of IL-2 secretion and bioactivity in culture supernatants (Fig. S2d).

Together, these results suggest that the canonical branch point in segment 8 is dispensable to vector performance. Moreover, its inclusion has, at most, a modest negative effect on viral replication, possibly due to interference with context-specific splicing dynamics. Overall, these results are consistent with the use of non-canonical splicing pathways for influenza A virus mRNAs, which require slightly different *cis*-acting elements compared to those used by most host mRNAs (5, 34, 35).

### Novel ΔNS1 vectors are attenuated in IFN-competent systems but support robust cytokine expression *in vitro* and *in vivo*

We demonstrated that the novel vector design carrying the IL-2 cassette replicated efficiently and induced robust IL-2 secretion in MDCK-NS1 cells. However, given that IAV-ΔNS1 vectors are known to be attenuated in IFN-competent systems, we next evaluated the vector’s ability to induce cytokine expression in these systems, which is critical for assessing its potential in the clinic.

**Fig 2:**
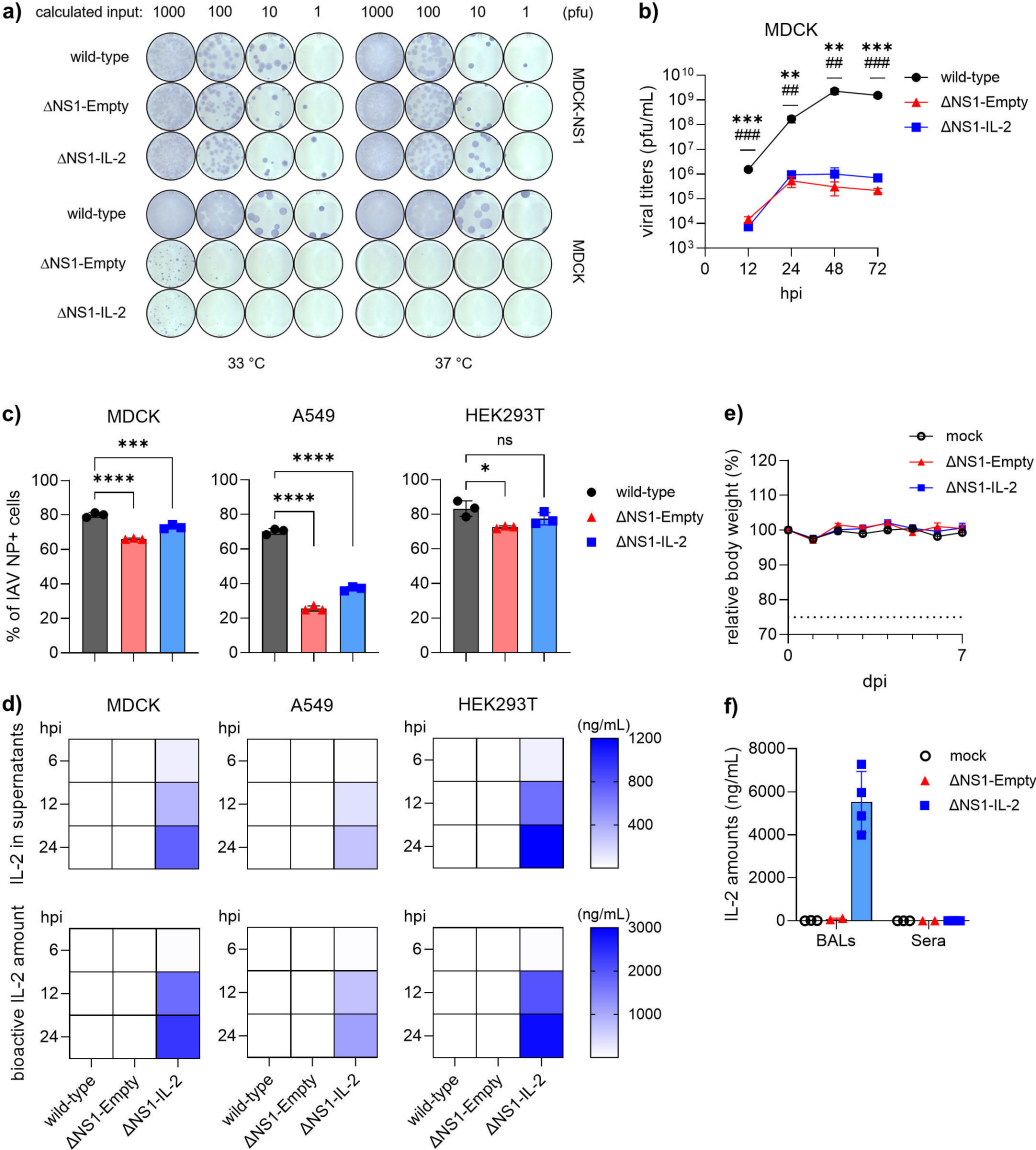
Novel IAV-ΔNS1 vectors induce notable viral antigen expression and vector-sourced IL-2 secretion in IFN-competent systems. (**a**) Representative images of plaque assays for pre-estimated doses of indicated viruses at 33°C or 37°C on MDCK or MDCK-NS1 cells. Plaques were stained by standard immuno-staining against IAV NP protein. (**b**) Multi-cycle growth curve analysis for the indicated viruses in MDCK cells. Data are depicted as mean ± SD (*n* = 3). Representative data from two independent experiments are shown. * and # indicate statistically significant differences between wild-type and vector control, or between wild-type and the IL-2-carrying vector, respectively. (**c and d**) MDCK, A549, and HEK293T cells were infected with the indicated viruses at an MOI of 1. (**c**) At 24 hpi, infection rates were quantified by flow cytometry analysis for IAV NP-expressing cells. Representative data from two independent experiments are depicted as mean ± SD (*n* = 3). One-way ANOVA was applied to test for statistical significance. *P* < 0.05. (**d**) IL-2 levels in culture supernatants and bioactivity of vector-sourced IL-2 were measured at the indicated time points by sandwich ELISA and HEK-Blue CD122/CD132 cells, respectively. Representative data from two independent experiments are depicted as median (*n* = 3). (**e and f**) BALB/c mice were intranasally inoculated with the vectors at a dose of 1 × 10^5^ pfu/animal or mock controls. (**e**) Animals were monitored for weight loss up to 7 days post-infection (*n* = 5). The dotted line indicates the corresponding human endpoint (75% of initial body weight). Data are depicted as mean ± SD. (**f**) IL-2 levels measured by sandwich ELISA in bronchoalveolar lavages (BALs) and serum collected from mice inoculated with the indicated viruses or mock controls at 24 hpi. Data are depicted as mean ± SD. Student’s *t*-test was used to test for statistical significance unless otherwise stated. *P* < 0.05. ng, nanogram; mL, milliliter; and hpi, hours post-infection.

Indeed, multi-cycle plaque assays at 33°C and 37°C showed minimal or no plaque formation for the ΔNS1 vectors in non-transduced MDCK cells (lacking NS1 expression), whereas comparable plaque sizes and numbers were observed between wild-type viruses and the vectors in MDCK-NS1 cells at the same inoculum (Fig. 2a). To further assess the replicative capacity of the vectors in IFN-competent systems, MDCK cells were infected with wild-type, ΔNS1-IL-2, or ΔNS1-Empty viruses at an MOI of 0.02. Quantification of infectious viral particles by plaque assay demonstrated a 2–3 log reduction in viral titers for the ΔNS1 vectors throughout the infection (Fig. 2b), confirming the attenuated phenotype. To determine whether this attenuation impaired the infection efficiency or cytokine production, we infected several IFN-competent cell lines (MDCK, A549, and HEK293T) with PR/8 wild-type, ΔNS1-Empty, or ΔNS1-IL-2 viruses at an MOI of 1. Flow cytometric analysis of NP-positive cells revealed a modest but significant reduction in infectivity for the ΔNS1 vectors compared to wild-type virus, with the degree of reduction varying by cell type (~10% in MDCK and HEK293T; ~50% in A549, Fig. 2c). Consistent with these observations, time-course measurements of IL-2 in cell culture supernatants demonstrated robust cytokine secretion, with concentrations proportional to infection rates across cell types (Fig. 2d). We concluded that, despite clear attenuation in the replicative cycle, the vectors were capable of efficiently infecting and delivering the transgene in IFN-competent systems.

Given the promising results *in vitro*, we sought to assess the infectivity and the transgene expression *in vivo*. To this end, BALB/c mice (7–8 weeks old) were intranasally inoculated with 10⁵ pfu of either ΔNS1-Empty or ΔNS1-IL-2 viruses. As expected, the ΔNS1 viruses were attenuated *in vivo*, with no clinical signs or weight loss observed up to 7 days post-infection (Fig. 2e). At 24 hours post-infection, bronchoalveolar lavage fluid (BALF) and serum were collected. IL-2 was detected exclusively in the BALF of ΔNS1-IL-2-infected mice, indicating that the secretion was vector sourced and localized to the respiratory tract. No IL-2 was detected in serum within the range tested (>50 pg/mL) (Fig. 2f).

These findings suggest that the re-engineered virus retains the ability to initiate primary infection and drive functional cytokine secretion both *in vitro* and *in vivo* settings.

### Re-engineered ΔNS1 vector exhibits functional versatility in transgene choice

**Fig 3:**
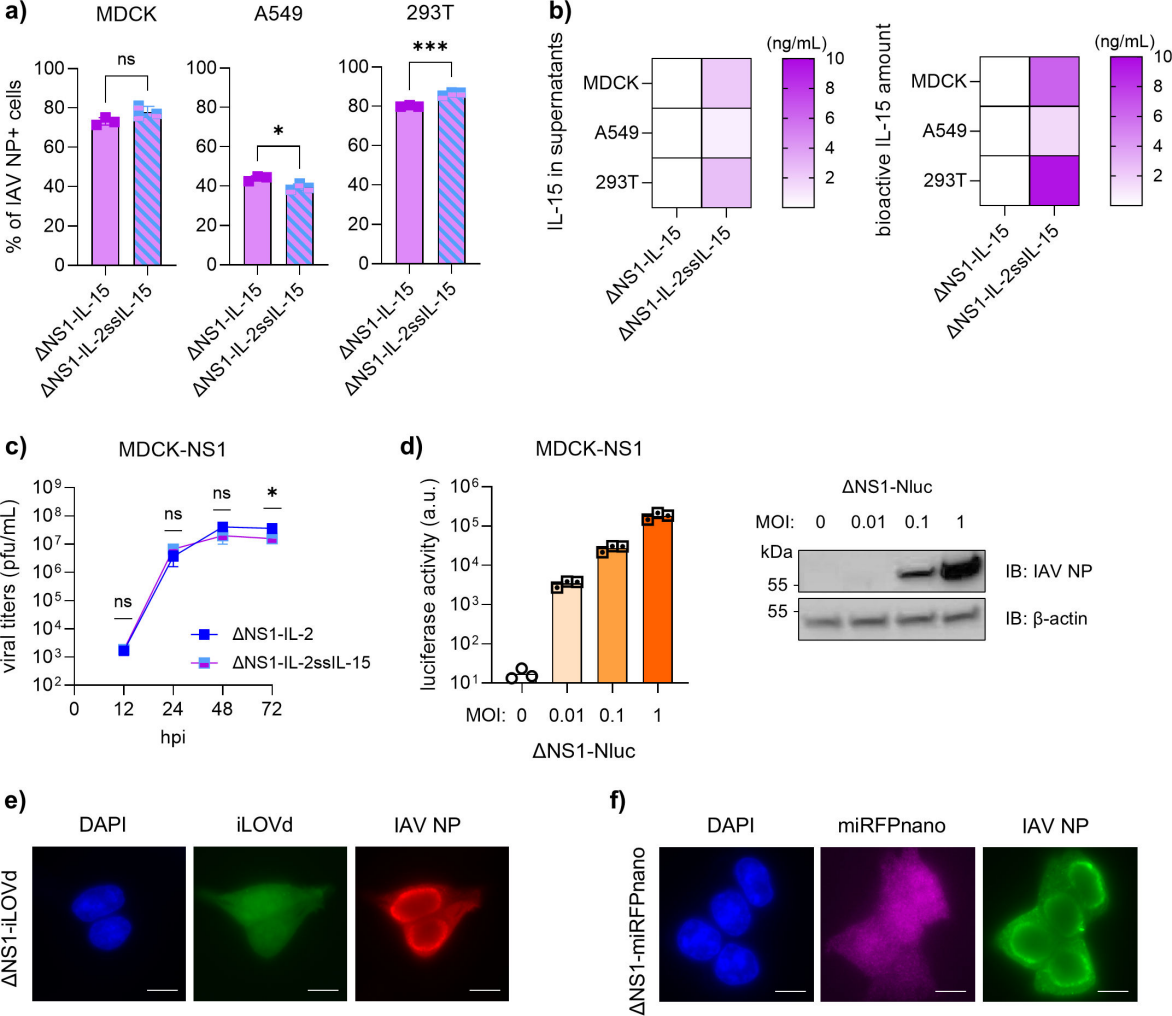
The vector can accommodate a diverse array of transgenes, highlighting the versatility of the platform. (**a and b**) MDCK, A549, and HEK293T cells were infected with indicated viruses at an MOI of 1. (**a**) At 24 hpi, infection rates were quantified by flow cytometry analysis for IAV NP-expressing cells. Representative data from two independent experiments are depicted as mean ± SD (*n* = 3). (**b**) IL-15 levels in culture supernatants and bioactivity of vector-sourced IL-15 were measured at 24 hpi by sandwich ELISA and HEK-Blue CD122/ CD132 cells, respectively. Representative data from two independent experiments are depicted as median (*n* = 3). (**c**) Multi-cycle growth curve analysis for the indicated viruses in MDCK-NS1 cells. Data are depicted as mean ± SD (*n* = 3). Representative data from two independent experiments are shown. (**d**) Representative data for luciferase activity measured at 24 hpi in MDCK-NS1 cells infected with IAV-ΔNS1 vector carrying nanoluciferase gene at different MOIs. Data are depicted as mean ± SD. Western blot images acquired from the same lysates were blotted against a loading control (β-actin) and IAV NP protein. (**e and f**) Representative microscopy images of HEK293T cells infected with IAV-ΔNS1 vector carrying iLOVd (**e**) and miRFPnano (**f**) at 8 hours post-infection. DAPI is used for nuclear staining. Student’s *t-*test was used to test for statistical significance unless otherwise stated. *P* < 0.05. ng, nanogram; mL, milliliter; hpi, hours post-infection; pfu, plaque-forming unit; au, arbitrary unit; IB, immunoblot; and kDa, kilodalton.

We next assessed the capacity of the novel ΔNS1 platform to accommodate other cytokines and reporter genes. As an example, we incorporated IL-15, another cytokine that plays a pivotal role in T-cell and NK cell activation. The native signal peptide sequence within the human IL-15 ORF is sub-optimal for secretion; however, replacing the signal peptide with that of IL-2 significantly enhances its secretion *in vitro* (36). Accordingly, we generated two constructs expressing IL-15 with either its native signal peptide (ΔNS1-IL-15) or with that of IL-2 (ΔNS1-IL-2sp-IL-15). Indeed, the ΔNS1-IL-15 construct failed to induce detectable IL-15 secretion in cell culture supernatants upon infection (Fig. 3b), although infection rates were similar between the constructs in all cell lines tested (Fig. 3a). Notably, the ΔNS1-IL-2sp-IL-15 construct supported robust IL-15 secretion (Fig. 3b). We concluded that the vector can successfully carry and express other cytokines, and their secretion can easily be enhanced through signal peptide engineering.

Next, we evaluated whether the incorporation of a different transgene affected the replication kinetics of the vector. Multi-cycle replication assays in MDCK-NS1 cells showed no significant difference in viral growth between vectors carrying IL-2 or IL-2sp-IL-15, except 72 hours post-infection, where modest but significant differences were observed (Fig. 3c). We concluded that replacing the transgene did not substantially alter viral fitness, further supporting the platform’s flexibility in transgene selection.

To explore the potential of the vector for reporter gene expression, we incorporated the nanoluciferase gene in the ΔNS1 background. Infection of MDCK-NS1 cells with serial 10-fold dilutions of the virus resulted in a dose-dependent increase in luciferase signal, which correlated with viral protein expression as confirmed by luciferase assay and Western blot, respectively (Fig. 3d). We then tested two additional compact fluorescent reporter genes: miniSOG (a fluorescent LOV domain-derived protein, LOVd) (37) and miRFP670nano (38), which were selected based on size compatibility with the vector. Recombinant viruses carrying these reporters were successfully rescued using reverse genetics. Upon infection at an MOI of 1, HEK293T cells were fixed and stained for NP protein at 8 hours post-infection. Fluorescence microscopy confirmed co-expression of both the reporter and NP proteins in the infected cells (Fig. 3e and f).

Together, these results highlight the versatility of the re-engineered ΔNS1 vector in delivering a wide range of functional proteins and reporter genes.

## DISCUSSION

Several studies have explored integrating transgenes into segment 8 of IAVs, particularly in ΔNS1 backgrounds (27–31). While such insertions are generally well tolerated in terms of size, the segment structure presents distinct challenges, largely due to the overlap between the NS1 and NEP ORFs and their shared N-termini. A common strategy in ΔNS1 vectors is expressing the transgene as a 2A fusion, or similar protease links, with the NEP protein (27, 31). Although this approach effectively supports the transgene expression, it raises concerns for premature accumulation of NEP protein due to the *cis*-placement of the NEP cassette, thereby disrupting the temporal regulation of NEP levels. Additionally, expression via 2A fusion may lead to inefficient cleavage or steric hindrance of the transgene, particularly for secretory proteins. Indeed, the construct in which IL-2 was fused to NEP via a porcine teschovirus-1 2A sequence failed to yield detectable cytokine secretion (Fig. S1a). In contrast, alternative designs place the gene cassette in the intronic region in lieu of the NS1 ORF, preserving splicing competence, which in turn maintains proper control of NEP protein levels. In such designs, transgene expression is achieved by introducing a ribosomal stop/start signal downstream of the NS1 N-terminus (28–30). However, these vectors often require complex genetic modifications to ensure sufficient expression and may result in the accumulation of unintended NS1 peptide fragments and present genomic stability issues, limiting their clinical utility.

In this study, we developed a re-engineered segment 8 design that is the first to fully decouple the NEP ORF from the N-terminal coding region in a splicing-competent background (Fig. 1a). This innovation frees the intronic region for transgene insertion and allows translation initiation from the transgene ORF, with minimal impact on the splicing of segment 8 (Fig. 1c and d). Using human IL-2 as a model transgene, we demonstrated robust secretion of bioactive cytokine from infected cells (Fig. 1f and g), confirming efficient transgene incorporation. Importantly, vectors carrying the transgene achieved viral titers comparable to wild-type virus (Fig. 1e). Furthermore, no mutations in the segment sequence were observed after 10 consecutive passages (Table S1), and IL-2 secretion was maintained across all passages (Fig. 1h), demonstrating high genetic stability. Our findings establish a streamlined, functionally efficient platform for transgene expression in IAV-ΔNS1 vectors, particularly well-suited for secretory proteins and those requiring N-terminal modifications.

Splicing efficiency is a critical determinant of NEP expression. Previous studies have shown that native NS segment splicing occurs at low efficiency, approximately at a ratio of 1:10 (5, 12, 39). In certain ΔNS1 designs, where the transgene is placed in a 2A fusion with NEP, NEP mRNA abundance parallels the segment copy number (27, 31), resulting in excess NEP protein. In other designs, where splicing regulatory elements are retained, the temporal dynamics of NEP expression are restored (28–30). However, transgene expression levels are relatively reduced due to stop/start signals, causing inefficient translation initiation. In our experiments, the re-engineered vector exhibited a splicing ratio of approximately 1:14 (6.6%), closely resembling that of the wild-type virus (6.9%) (Fig. 1c) at early time points. Notably, relative NEP expression was slightly reduced in the IL-2-carrying vector (Fig. 1d), which we speculate that may result from the extended distance to the translation start site or a change in Kozak strength. Despite this, viral replication kinetics of the vector remained comparable to wild-type, while maintaining robust transgene expression (Fig. 1e through g). Interestingly, restoring the canonical branch point and the complete polypyrimidine tract resulted in a modest increase in splicing efficiency; however, no corresponding impact on viral growth or cytokine secretion was observed (Fig. S2a through d). This suggests that cryptic or compensatory elements within the transgene sequence may facilitate efficient spliceosome assembly. We cannot rule out, however, that the branch point may be dispensable for the NS segment splicing, with its function potentially compensated for by other RNA-binding proteins. This remains speculative and beyond the scope of this study.

We further demonstrated the broad applicability of this platform by integrating additional transgenes, including human IL-15 and various reporter genes. As previously reported, the native IL-15 signal peptide was insufficient to induce cytokine release in the supernatants, but this was rescued by replacing it with IL-2 signal peptide sequence (Fig. 3a and b). Yet, both genes were easily incorporated into the platform. As also shown in comparison to IL-2-carrying construct, the choice of the transgene had minimal impact on viral replication kinetics (Fig. 3c). We also tested other reporter transgenes, such as nanoluciferase, and compact fluorescent reporters like miniSOG and miRFPnano670. The vector backbone successfully accommodated diverse payloads (Fig. 3d through f). We observed that insert size may be a limiting factor for the platform, as vectors with larger cassettes, such as mCherry or GFP, were difficult to stably rescue (data not shown), suggesting an upper size threshold or sequence-specific limitation.

Flu accounts for 3–5 million cases of severe illness and up to 650,000 deaths annually, especially in high-risk groups, including children, the elderly, and immunocompromised patients (40). Despite its limitations, seasonal vaccination against flu remains the most effective method for preserving public health. IAV-ΔNS1 vectors, which have gained attention as live attenuated vaccine platforms, are attenuated *in vitro* and *in vivo* but can be produced in high quantities in interferon signaling-deficient systems or where NS1 protein is supplied exogenously (18–21) Previous studies have demonstrated favorable safety and immunogenicity profiles of IAV-ΔNS1 vectors in both animal models and early clinical trials (18–20, 22, 24–26). The re-engineered vector provides a significant advancement in vaccine development for IAV-ΔNS1 vectors, as it can be used for localized therapeutic delivery or vaccine adjuvant enhancement, particularly for immunocompromised populations. Furthermore, the system can be customized to modulate specific immune cell subsets and their responses by incorporating alternative chemokines and cytokines as transgenes.

In conclusion, our study presents a robust and flexible platform for stable and efficient transgene expression within the NS segment of IAV-ΔNS1 vectors. This design simplifies vector construction, preserves viral fitness, prevents generation of secondary protein fragments, and supports the expression of diverse immunomodulatory and reporter genes. These features make the platform a promising tool for applications in vaccine development, therapeutic delivery, and fundamental virology research.

## MATERIALS AND METHODS

### Cells and culture conditions

Human embryonic kidney HEK293T cells (ATCC CRL-3216) and A549 lung epithelial cells (ATCC CCL-185) were obtained from the American Type Culture Collection. Wild-type MDCK cells and MDCK cells stably expressing the NS1 gene (MDCK-NS1) were kindly provided by Dr. Umut Karakus and Prof. Adolfo García-Sastre, respectively. Briefly, MDCK cells were transduced with a construct expressing the NS1 protein of influenza A virus strain A/Puerto Rico/8/1934 fused to GFP, as previously described (41). HEK-Blue CD122/132 cells, engineered to monitor the bioactivity of human IL-2 and IL-15, were purchased from InvivoGen.

All cell lines were cultured in complete growth medium consisting of Dulbecco’s Modified Eagle Medium with 4.5 g/L glucose, L-glutamine, and sodium pyruvate (Corning), supplemented with 10% heat-inactivated fetal bovine serum (Corning) and 1% Penicillin-Streptomycin (100×; Corning). Cells were maintained at 37°C in a humidified incubator with 5% CO_2_. For antibiotic selection, MDCK-NS1 cells were cultured in complete medium supplemented with Hygromycin B (Invitrogen) at 0.1 mg/mL. HEK-Blue CD122/132 cells were maintained in the complete medium additionally supplemented with Normocin (100 µg/mL), 1× HEK-Blue Selection, and Puromycin (1 µg/mL; Corning).

### Plasmids and vector construction

Ambisense expression plasmids (pDZ) encoding genomic segments 1–8 of influenza A virus strain A/Puerto Rico/8/1934 (PR/8) were kindly provided by Prof. Adolfo Garcia-Sastre. The re-engineered plasmid backbone carrying transgenes was derived from the pDZ vector expressing PR/8 segment 8 (see Fig. 1a). To generate the 5′ portion of the modified segment, complementary oligonucleotides (primers 1F and 1R; Table S2) encoding the 3′ untranslated region, packaging signals, and splicing donor motif were denatured at 95°C and allowed to anneal through gradual lowering of temperature at a rate of −1°C per second. The annealed oligos were purified using the Cycle Pure Kit (E.Z.N.A., Omega). To prevent unwanted translation initiation, the start codon (ATG) of the NS1 ORF within the oligos was mutated to TTG (highlighted bold, see Table S2).

In parallel, the downstream fragment covering the splicing acceptor site, nuclear export protein ORF, and 5′ packaging signal was PCR-amplified from a pDZ-segment 8-ΔNS1 construct (21) using Phusion High-Fidelity DNA polymerase (NEB, USA) with primers 2F and 2R (see Table S2), except ΔNS1-Empty construct, which was cloned with 2.1F (see Table S2). Within the fragment, the splicing acceptor site was re-positioned upstream of the full-length NEP ORF, thus liberating the ORF dependency of NEP on NS1 ORF. The PCR product was size separated by agarose gel electrophoresis and purified using the Gel Extraction Kit (E.Z.N.A., Omega).

Human IL-2 (NM_000586) and IL-15 (NM_172174) complementary DNAs (cDNAs) were cloned from commercially available plasmids (RC210013 and RC219294, respectively; Origene). An alternate version of IL-15 containing the IL-2 signal sequence (IL-2ssIL-15) was generated in-house using standard cloning methods, based on previous reports (36). Additional transgenes included nanoluciferase (NLuc), cloned from a plasmid generously provided by Prof. Luis Martinez-Sobrido; miniSOG, cloned from miniSOG-N1 (a gift from Michael Davidson; Addgene plasmid #54824) (37); and miRFP670nano, cloned from pcDNA-miRFP670nano (a gift from Vladislav Verkhusha; Addgene plasmid #127443) (38). Transgenes were amplified from source plasmids using Phusion High-Fidelity DNA polymerase (NEB) with primers flanked by complementary nucleotides to the adjacent upstream and downstream segments (see Table S2 for each transgene). PCR products were gel purified (E.Z.N.A., Omega). The final constructs—comprising the annealed upstream fragment, the transgene, and the downstream NEP-containing fragment—were assembled via overlap extension PCR using primers 3F and 3R (Table S2). Assembled inserts were cloned into SapI-digested pDZ vector backbone using In-Fusion cloning (Takara Bio), following the manufacturer’s protocol. Cloned plasmids were transformed into Stellar cells (Takara Bio) and plated on LB-agar containing ampicillin (50 µg/mL). Selected colonies were cultured in LB broth, plasmids were isolated using the Mini-prep Plasmid Isolation Kit (E.Z.N.A., Omega), and all insert sequences were confirmed by Sanger sequencing (Eton Bioscience).

The final segment design, in 5′–3′ orientation in pDZ backbone, included the 3′ non-coding region (NCR) of segment 8, the partial NS1 ORF, including the splicing donor motif (ATG > TTG), the transgene (Empty, IL-2, IL-15, IL-2ssIL-15, NLuc, miniSOG, or miRFP670nano), the splicing acceptor motif, the complete NEP ORF, and the 5′ NCR of segment 8 (Fig. 1a). For selected transgenes, the branch point and polypyrimidine tract were restored via PCR using additional flanking primers (IL-2+BP-F and IL-2+BP-R, see Table S2). Additionally, the ΔNS1-IL-2-2A-NEP construct was generated based on a previously reported design (31) using the primers listed in Table S2. All oligonucleotides were synthesized by Eton Bioscience.

### Rescue of viral infectious clones and preparation of viral stocks

Co-cultures of HEK293T and MDCK-NS1 cells were seeded in a 6-well plate format at a density of 400,000 cells per well each in complete growth media. The following day, cells were transfected with 1 µg of each ambisense pDZ plasmid encoding influenza A/Puerto Rico/8/1934 segments PB2, PB1, PA, HA, NP, NA, and M, along with 1 µg of pDZ-∆NS1-Empty, pDZ-∆NS1-IL-2, pDZ-∆NS1-IL-2-2A-NEP, pDZ-∆NS1-IL-15, pDZ-∆NS1-IL-2ss-IL-15, pDZ-∆NS1-NLuc, pDZ-∆NS1-miniSOG, pDZ-∆NS1-miRFP670nano, or pDZ-∆NS1-IL-2+BP plasmids, using LT1 transfection reagent (Mirus) according to the manufacturer’s instructions. After an overnight incubation at 37°C with 5% CO_2_, cells were washed once with 1× phosphate-buffered saline (PBS) without Ca²^+^ and Mg²^+^ (Gibco), and the medium was replaced with infection medium, following the recipe described in reference 42, supplemented with TPCK-treated trypsin (1 µg/mL; Sigma-Aldrich). Cells were incubated at 33°C with 5% CO_2_ for an additional 48 hours. Supernatants were collected and clarified by centrifugation (10,000 ×* g* for 5 minutes) to remove cell debris. Cleared supernatants were tested for the presence of viral particles using a hemagglutination assay, as described elsewhere (42, 43). Viral RNA was extracted using the Viral RNA Kit (E.Z.N.A., Omega) according to the manufacturer’s protocol. Segment 8 was amplified from the viral RNAs via segment-specific RT-PCR using the SuperScript III One-Step RT-PCR System with Platinum Taq DNA Polymerase (Invitrogen) and primers 4F and 4R (see Table S2). Amplicons were confirmed by Sanger sequencing (Eton Bioscience). Infectious clones were quantified using standard plaque assays, as detailed previously (44). Viruses were further amplified in large batches on MDCK-NS1 cells from plaque-purified clones and stored at –80°C in aliquots for downstream applications. Whole-genome sequencing of viral stocks and passaged isolates was performed and analyzed using Oxford Nanopore Technologies and a related bioinformatics pipeline, as further detailed below.

### Quantification of splicing by real-time quantitative PCRs

HEK293T cells were seeded at a density of 2.5 × 10^5^ cells/well in a 24-well format and cultured overnight. The next day, cells were infected with recombinant IAV carrying wild-type segment 8, ΔNS1-IL-2, or ΔNS1-IL-2+BP at an MOI of 1. At the indicated time points (6, 12, and 24 hours post-infection), cells were washed once with 1× PBS, and total RNA was extracted using E.Z.N.A. HP Total RNA Kit (Omega Bio-tek) following the manufacturer’s guidelines. Resultant RNAs were reverse transcribed into complementary DNA with the Omniscript RT Kit (Qiagen) supplemented with Oligo(dT) primers (Invitrogen) according to the manufacturer’s instructions. Then, cDNAs were quantified by SYBR green-based qPCR using the LightCycler 480 SYBR Green I Master mix and specific primers (listed in Table S2) on a LightCycler 480 II qPCR system (Roche). The copy number of each mRNA was calculated based on the standard curve generated using 10-fold serial dilutions of pure plasmid solutions containing the respective coding sequences (pCAGGS-NS1, pCAGGS-NEP, and pDZ-ΔNS1-IL-2).

### Whole-genome sequencing using Oxford Nanopore Technologies

Library preparation from viral RNA extracts was performed following previously described methodology (45). Briefly, viral segments were amplified from 100–200 ng of each viral RNA sample by RT-PCR using targeted primers—Tuni 12 (5′-ACGCGTGATCAGCAAAAGCAGG-3′), Tuni 12.4 (5′-ACGCGTGATCAGCGAAAGCAGG-3′), and Tuni 13 (5′-ACGCGTGATCAGTAGAAACAAGG-3′)—as previously described, with the SuperScript IV One-Step RT-PCR System (Invitrogen). Amplicons were purified using AMPure XP beads (Beckman Coulter), quantified using Qubit (Thermo Fisher Scientific), and pooled into a sequencing library at equimolar concentrations (200 fmol per sample). Barcoding and adapter ligation were performed using the Oxford Nanopore SQK-NBD114.24 kit, following the manufacturer’s instructions. The pooled library was further purified with AMPure XP and loaded onto a MinION flow cell (R10.4.1, Oxford Nanopore Technologies). Real-time basecalling, adapter trimming, and demultiplexing were performed using MinKNOW (Oxford Nanopore Technologies). Processed reads were aligned to reference sequences using Minimap2 in Geneious Prime, and SNPs with a variant frequency ≥25% were identified.

### Plaque assays and viral passages

For multi-cycle growth curves, MDCK cells or MDCK-NS1 cells were infected with selected viruses at a multiplicity of infection of 0.002 for 1 hour at 37°C in 5% CO_2_. After incubation, the viral inoculum was removed and replaced with infection medium prepared as previously described (42). Supernatants were collected at specified time points and clarified by centrifugation at 10,000 × *g* for 5 minutes at 4°C. Aliquots were frozen at –80°C for downstream applications. Infectious titers were determined by standard plaque assays on MDCK-NS1 cells in a 12-well format, followed by counterstaining with crystal violet (44). Where indicated, plaque assays performed on MDCK wild-type or MDCK-NS1 cells were visualized by IAV-specific immunostaining, as described in reference 44 with slight modifications. Briefly, following standard plaque assay, cells were fixed with 4% formaldehyde for 1 hour and permeabilized with 0.1% Triton X-100 in PBS for 15 minutes. Wells were then blocked with 3% bovine serum albumin (BSA) in PBS for 30 minutes and incubated overnight with primary antibody (anti-IAV sera) in PBS containing 0.05% Tween 20. The following day, cells were washed twice with 1× PBS and incubated for another hour with a horseradish peroxidase (HRP)-conjugated anti-rabbit IgG secondary antibody. After several washes, plates were developed using TrueBlue substrate (Thermo Scientific) following the manufacturer’s protocol.

For viral passaging, MDCK-NS1 cells were seeded at full confluency in a 6-well plate format. Freshly rescued IAV-ΔNS1-Empty or IAV-ΔNS1-IL-2 viruses were added at a 1:2,000 dilution in triplicate under blinded conditions. After 72 hours of incubation, supernatants were collected, clarified through centrifugation (10,000 × *g* for 5 minutes), and either used immediately or stored for subsequent passages. This process was repeated for 10 passages. Viral RNA from the 10th passage was extracted, prepared for library preparation, and sequenced using Oxford Nanopore Technologies to identify adaptive mutations, following the procedure described above.

### Enzyme-linked immunosorbent assay and cytokine bioactivity assays

MDCK-NS1, MDCK, HEK293T, or A549 cells were seeded in complete growth medium at a density of 400,000 cells per well in a 6-well plate format. The next day, after washing with PBS, cells were mock-infected or infected with the indicated viral vectors for 1 hour at 37°C in 5% CO_2_. Then, the viral inoculum was removed, replaced with infection medium, and the cells were further incubated until the specified time points (e.g., 6, 12, 24, or 72 hours post-infection). At each time point, supernatants were collected, clarified by centrifugation to remove cellular debris, and heat-inactivated at 56°C for 30 minutes to neutralize infectious particles. IL-2 and IL-15 concentrations in the supernatants were measured using the respective OptEIA Human IL-2 and IL-15 ELISA Sets (BD Biosciences), according to the manufacturer’s instructions. Plates were developed using 3,3′,5,5′-Tetramethylbenzidine (BD), and the reaction was stopped with 1 N sulfuric acid (Fisher). Absorbance was measured at 450 nm using a BioTek Synergy Neo2 Hybrid Multi-Mode Microplate Reader (Agilent). Cytokine concentrations were calculated by fitting sample values to standard curves using four-parameter logistic regression in GraphPad Prism.

To assess cytokine bioactivity, HEK-Blue CD122/132 reporter cells (InvivoGen), which respond to IL-2 and IL-15 signaling by STAT5 activation and consequent induction of secreted alkaline phosphatase (SEAP) production, were used following the manufacturer’s protocol. Briefly, 50,000 reporter cells per well were incubated with heat-inactivated supernatants in a 96-well plate for 24 hours at 37°C. The following day, supernatants were mixed with Quanti-Blue detection reagent, and SEAP activity was measured at 650 nm using the microplate reader. Recombinant human IL-2 and IL-15 were included in parallel to generate dose–response curves for bioactivity quantification.

### Flow cytometry and fluorescent microscopy

For flow cytometric analysis, MDCK, A549, or HEK293T cells were seeded at a density of 400,000 cells per well in a 6-well plate and infected at an MOI of 1, as described above. At 24 hours post-infection, cells were washed twice with 1× PBS and detached using 0.25% Trypsin-EDTA (Gibco). Cell suspensions were centrifuged at 500 × *g* for 5 minutes, washed once with 1× PBS, and centrifuged again. Cell pellets were fixed using Fixation Buffer (BioLegend) for 30 minutes at room temperature. Following fixation, cells were washed with Cell Staining Buffer (BioLegend), permeabilized with Permeabilization Buffer (BD Biosciences), and stained for influenza A virus nucleoprotein using Alexa Fluor 488-conjugated HT103 antibody in Permeabilization Buffer (BD Biosciences) for 1 hour at room temperature, following the manufacturer’s instructions. Cells were then washed twice with Cell Staining Buffer (BioLegend) and resuspended in 1× PBS. Flow cytometry data were acquired using a Gallios Flow Cytometer (Beckman Coulter) or an Attune NxT Flow Cytometer (Thermo Fisher Scientific) and analyzed for NP-positive populations using FlowJo software.

For fluorescence microscopy, HEK293T cells were seeded at a density of 70,000 cells per well in a glass-bottom 24-well plate. After standard infection with ΔNS1-LOVd (miniSOG) or ΔNS1-miRFP670nano at an MOI of 1, cells were fixed at 8 hours post-infection with 4% formaldehyde for 1 hour at room temperature after two 1× PBS washes. Cells were then permeabilized with 0.1% Triton X-100 for 15 minutes, washed again, and blocked with 3% BSA in PBS. Primary staining was performed using mouse anti-influenza A virus NP antibody (HT103) overnight at 4°C. The following day, cells were washed twice with PBS and incubated with the appropriate secondary antibody, Alexa Fluor 594-conjugated anti-mouse IgG for LOVd-expressing viruses, or Alexa Fluor 488-conjugated anti-mouse IgG for miRFP670nano-expressing viruses, and DAPI for nuclear staining for 1 hour at room temperature. After three final PBS washes, fluorescent images were acquired using an EVOS FL Auto 3000 imaging system (Thermo Fisher Scientific).

### Western blot and luciferase assay

HEK293T cells were seeded at a density of 400,000 cells per well in a 6-well plate format on poly-L-lysine-coated plates. The next day, cells were mock-infected or infected with the indicated viruses at an MOI of 1 following the standard infection protocol. After infection, cells were washed once with 1× PBS and lysed in 2× Sample Buffer (Bio-Rad) supplemented with 2-mercaptoethanol, diluted in RIPA buffer (Sigma), and supplemented with 1% sodium dodecyl sulfate and cOmplete protease inhibitor cocktail (Roche). Lysates were transferred to microcentrifuge tubes and boiled at 100°C for 5 minutes. Following centrifugation at 10,000 × *g* for 10 minutes, supernatants were loaded onto 4%–20% Mini-PROTEAN TGX precast gels (Bio-Rad) and electrophoresed alongside a molecular weight marker at 100 V for approximately 90 minutes. Proteins were transferred onto 0.2 μm nitrocellulose membranes using the Trans-Blot Turbo Transfer System (Bio-Rad) according to the manufacturer’s protocol. Membranes were blocked in 5% nonfat dry milk in Tris-buffered saline containing 0.1% Tween-20 and probed with the following primary antibodies: rabbit anti-influenza A virus NP (Genetex, GTX636247) for NP expression, rabbit anti-influenza A virus NS2/NEP (Genetex, GTX125952) for NS1 and NEP expression, and rabbit anti-β-actin (Genetex, GTX537675) as a loading control. HRP-conjugated donkey anti-rabbit IgG (Cytiva, NA934V) was used as the secondary antibody where applicable. Signal was developed using SuperSignal West Pico PLUS Chemiluminescent Substrate (Thermo Fisher Scientific), and membranes were imaged using high-contrast autoradiography film and a film developer.

For luciferase assays, HEK293T cells were seeded at a density of 100,000 cells per well in 24-well plates. The following day, cells were infected with either mock controls or ΔNS1-NLuc at varying MOIs in 10-fold dilutions. At 24 hours post-infection, luciferase activity was measured using the Nano-Glo Luciferase Assay System (Promega) according to the manufacturer’s instructions. Luminescence was recorded using the BioTek Synergy Neo2 Hybrid Multi-Mode Microplate Reader (Agilent). As matched controls for the assay, leftover cell lysates prepared in Passive Lysis Buffer (provided in the kit) were diluted with 2× Sample Buffer (Bio-Rad) supplemented with 2-mercaptoethanol and processed for Western blotting as described above. Membranes were blotted for NP expression and β-actin as a loading control.

### Mice

Female BALB/c mice (7–8 weeks old) were obtained from Jackson Laboratories and housed under specific pathogen-free conditions in a BSL-2 facility. Mice were acclimated for 7 days prior to experimentation under a 12-hour light/dark cycle, with *ad libitum* access to food and water throughout. For intranasal inoculations, mice were anesthetized via intraperitoneal injection of ketamine (100 mg/kg of body weight) and xylazine (5 mg/kg of body weight) in 100 μL sterile PBS. Deep anesthesia was confirmed by loss of the pedal withdrawal reflex, after which mice were inoculated intranasally with 40 μL of either sterile PBS (vehicle control) or an IAV-ΔNS1 vector. Animals were monitored for clinical signs and weight loss up to 7 days post-infection.

At 24 hours post-inoculation (i.e., the experimental endpoint), submandibular blood collections were performed as described previously (46). Blood was collected into MiniCollect CAT serum separator tubes and allowed to clot overnight at 4°C to minimize hemolysis. The following day, tubes were centrifuged to separate serum from cellular components, and serum was stored at –20°C for subsequent ELISA analysis, as previously described.

Bronchoalveolar lavage (BAL) was conducted immediately following euthanasia using a modified protocol based on Sun et al. (47). Briefly, mice were euthanized via intraperitoneal injection of pentobarbital (250 mg/kg of body weight) and positioned on a Styrofoam platform. The skin from the abdomen to the neck was opened to expose the thoracic cavity and trachea. Surrounding soft tissues were carefully removed, and a ~10 cm sterile sewing thread was passed underneath the trachea. A small incision was made using a 21G sterile needle, and a 22G × 1″ BD Angiocath I.V. catheter was inserted approximately 0.5 cm into the trachea. The inner cannula was removed, and the catheter was secured with a double knot using the sewing thread. To collect BAL fluid, 1 mL of sterile saline (0.9%) was gently instilled into the lungs through the catheter using a 1 mL syringe and aspirated after 10–20 seconds. This step was repeated two additional times. Retrieved fluids were collected in sterile screw-cap tubes, centrifuged to remove cellular debris, and supernatants were aliquoted and stored at –20°C until further use.

### Quantification and statistical analysis

Quantification (e.g., four-parameter logistic regression) and statistical analyses (unpaired Student’s *t*-test) were performed using GraphPad Prism version 10, as detailed in the corresponding figure legends. Data are presented as mean ± standard deviation, with *n* = 3 unless otherwise specified. Statistical significance was defined as *P* ≤ 0.05. Significance thresholds are indicated as follows: *P ≤ 0.05, **P < 0.01, ***P < 0.001, and ****P < 0.0001; ns, not significant.

## Data Availability

All data generated and analyzed during this study were produced by the authors and are provided in the article and its supplemental material. Additional source data are available from the corresponding authors upon reasonable request.

## SUPPLEMENTAL MATERIAL

Supplemental material jvi.00347-26-s0001.pdf Fig. S1 and S2; Tables S1 and S2.

ASM does not own the copyrights to Supplemental Material that may be linked to, or accessed through, an article. The authors have granted ASM a non-exclusive, world-wide license to publish the Supplemental Material files. Please contact the corresponding author directly for reuse.
