# Supplementary material for: Re-engineering segment 8 facilitates generation of a versatile live-attenuated influenza A virus vector platform for secretory protein delivery
Source: J Virol. 2026 Apr 21;100(5):e00347-26. doi: 10.1128/jvi.00347-26 (PMC13185603; doi:10.1128/jvi.00347-26)
Supplement: Supplemental material — Fig. S1 and S2; Tables S1 and S2. [file jvi.00347-26-s0001.pdf]

Supplementary Figure 1. The IAV-ΔNS1-2A-IL2 virus fails to induce detectable IL-2 secretion in culture supernatants upon infection. a) Schematic representation for Influenza A virus mRNA encoded by the segment 8 construct in which IL-2 was linked to NEP ORF through 2A fusion. Icon descriptions are provided in the figure. Not to scale. **b)** IL-2 ELISA results for supernatants collected from MDCK-NS1 cells infected with indicated viruses at an MOI of 1 at 24 hours post-infection. Representative data from two independent experiments are depicted as Mean ± SD (n=3). **mL:** milliliter.

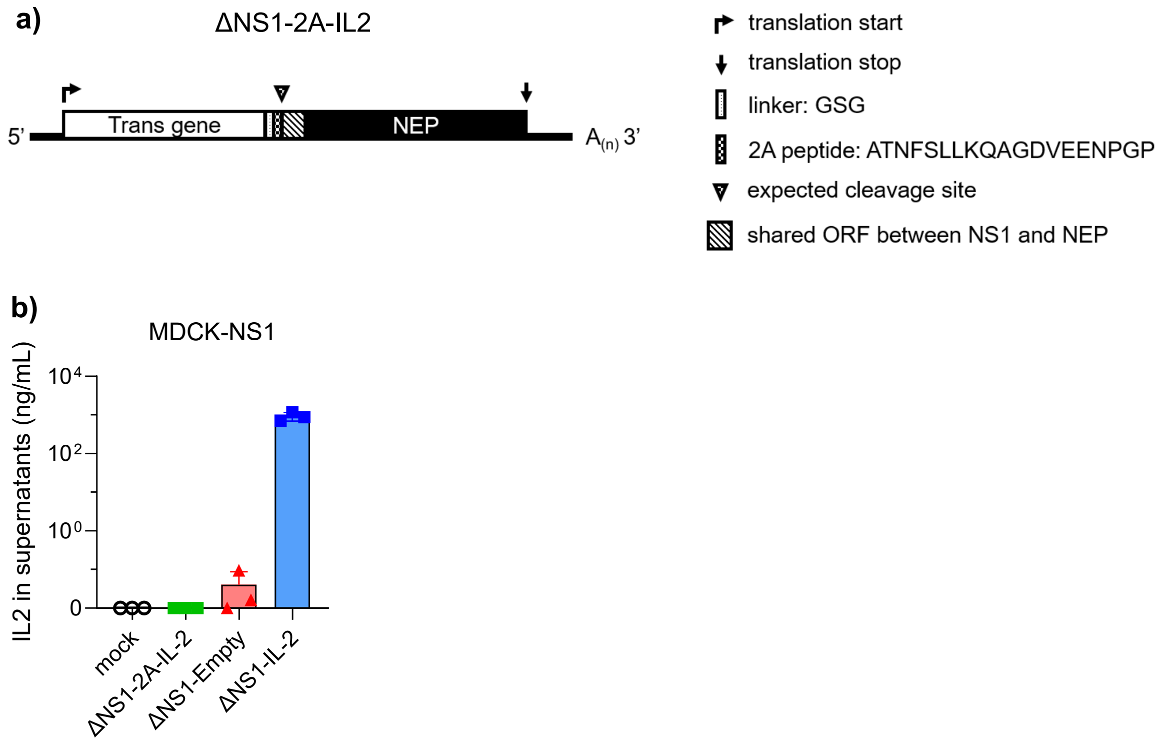

Supplementary Figure 2. Restoration of the canonical branch point did not impact the splicing competence, replication, or transgene delivery capacity of the engineered vector. a) Ratios of spliced versus total segment 8 mRNA copy numbers from HEK293T cells infected with viruses carrying ΔNS1-IL-2 or ΔNS1-IL-2 +BP segment 8 at an MOI of 1, quantified by qRT-PCR, at indicated time points. Representative data from two independent experiments are depicted as Mean ± SD (n=3). **b)** Western Blot images acquired from lysates of HEK293T cells infected with the indicated viruses or mock controls at 24 hours post-infection. Representative image is shown. **c)** Multi-cycle growth curve analysis for the indicated viruses in MDCK-NS1 cells. Data are depicted as Mean ± SD (n=3). Representative data from two independent experiments are shown. **d)** IL-2 ELISA and IL-2 bioactivity assay results for supernatants collected from MDCK-NS1 cells infected with indicated viruses at an MOI of 1 at 24 hours post-infection. Representative data from two independent experiments are depicted as Mean ± SD (n=3). Student's *t*-test is applied for significance test unless otherwise stated. $p<0.05$. **IB:** Immunoblot. **kDa:** kilodalton. **hpi:** hours post-infection. **pfu:** plaque forming unit. **mL:** milliliter. **ng:** nanogram.

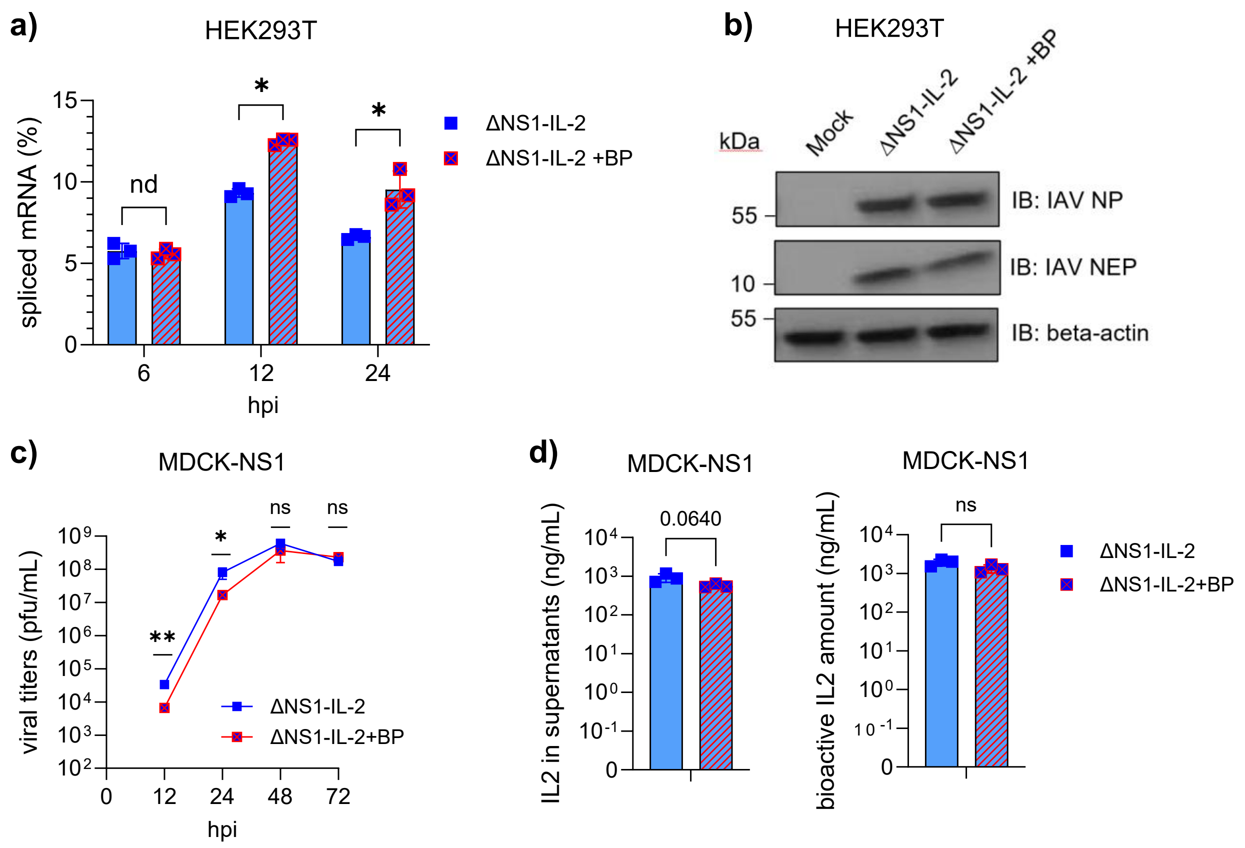

Supplementary Table 1. Mutations identified in the vector following 10 Serial Passages

virus	replicate	segment	nt position	change	variant frequency (%)	effect on translation
ΔNS1-IL-2	1	HA	446	C>A	64.0	aminoacid change in HA (S138R)
		NP	904	A>C	65.0	aminoacid change in NP (S287R)
	2	HA	446	C>A	67.0	aminoacid change in HA (S138R)
		NP	904	A>C	66.3	aminoacid change in NP (S287R)
	3	HA	446	C>A	61.5	aminoacid change in HA (S138R)
		NP	904	A>C	66.4	aminoacid change in NP (S287R)

Supplementary Table 2. List of primers used in this study

Primer	Application	Sequence (5'-3')
1F	Cloning	AGCAAAAGCAGGGTGACAAAGACATA**TTG**GATCCAAACACTGTGTCAAGCTTTCAGGTAGATTG
1R	Cloning	CAATCTACCTGAAAGCTTGACACAGTGTTTGGATC**CAA**TATGTCTTTGTCACCCTGCTTTTGCT
2F	Cloning	CTTCTCTTCCAGGAATGGATCCAAACAC
2.1F	Cloning	CACTGTGTCAAGCTTTCAGGTAGATTGACTTCTCTTCCAGGAATGGATCCAAACA
2R	Cloning	AGTAGAAACAAGGGTGTTTTTATTATTAAATAAGCTGA
IL-2-F	Cloning	ACTGTGTCAAGCTTTCAGGTAGATTGATGTACAGGATGCAACTCCTGTCTT
IL-2-R	Cloning	TCCTGGAAGAGAAGTTAAGTCAGTGTTGAGATGATGCTTTGAC
IL-15-F	Cloning	ACTGTGTCAAGCTTTCAGGTAGATTGATGAGAATTTCGAAACCACATTTGAGAAG
IL-15-R	Cloning	TTGGATCCATTCCTGGAAGAGAAGTTAAGAAGTGTTGATGAACATTTGGACAATATGTAC
IL-2ss-IL-15-F	Cloning	AGTCTTGCACTTGTCACAAACAGTAACTGGGTGAATGTAATAAGTGATTTGAAAAAAATT
IL-2ss-F	Cloning	ATGTACAGGATGCAACTCCTGTCTTGCATTGCACTAAGTCTTGCACTTGTCACAAACAGT
IL-2ss-R	Cloning	ACTGTTTGTGACAAGTGCAAGACTTAGTGCAATGCAAGACAGGAGTTGCATCCTGTACAT
NLuc-F	Cloning	ACTGTGTCAAGCTTTCAGGTAGATTGATGGTCTTCACACTCGAAGATTTCG
NLuc-R	Cloning	TTGGATCCATTCCTGGAAGAGAAGTTACGCCAGAATGCGTTCGCA
miniSOG-F	Cloning	ACTGTGTCAAGCTTTCAGGTAGATTGATGGAGAAGAGCTTCGTGATCACC
miniSOG-R	Cloning	TTGGATCCATTCCTGGAAGAGAAGTTAGCCGTCCAGCTGCAC
miRFP670nano-F	Cloning	ACTGTGTCAAGCTTTCAGGTAGATTGATGGCAAACCTGGACAAGATGC
miRFP670nano-R	Cloning	TTGGATCCATTCCTGGAAGAGAAGCTAGCTCTGCTGGATGGC
IL-2+BP-F	Cloning	TTTCACCATTGCCTTCTCTTCCAGGAATGGATCCAAAC
IL-2+BP-R	Cloning	TCCTGGAAGAGAAGGCAATGGTGAAATTAAGTCAGTGTTGAGATGATGCTTTGACAA
3F	Cloning	GACCTCCGAAGTTGGGGGGGAGCAAAAGCAGGGTGACAAAGACATATTG
3R	Cloning	TTGGGCCGCCGGGTTATTAGTAGAAACAAGGGTGTTTTTATTATTAAATAAGCTGAA
2A-NEP-F	Cloning	ATCTCAACACTGACTGGCAGCGGCGCGACCAAC
2A-NEP-R	Cloning	TTGGGCCGCCGGGTTATTAGTAGAAACAAGGGTGTTTTTATTA
IL-2-2A-F	Cloning	CTCCGAAGTTGGGGGGGAGCAAAAGCAGGGTGACAAAGACATAATGTACAGGATGCAACTCCTGTCTT
IL-2-2A-R	Cloning	GTCGCGCCGCTGCCAGTCAGTGTTGAGATGATGCTTTGACAAAAGGTAAT
4F	Segment 8 PCR	AGCAAAAGCAGGGTGACAAAGACA
4R	Segment 8 PCR	AGTAGAAACAAGGGTGTTTTTATTATTAAATAAGCTGAAACG
NS_All-F	qRT-PCR	TGGTTGATTGAAGAAGTGAGAC
NS_All-R	qRT-PCR	GAAAGTTCTTATCTCTTGCTCCA
NS_Spliced-F	qRT-PCR	GCAAAAGCAGGGTGACAAAGAC
NS_Spliced-R	qRT-PCR	GTCTCACTTCTTCAATCAACCAT
